# Holding Complex Spaces Through Relational Work: A Qualitative Descriptive Study of Co‐Design Facilitation

**DOI:** 10.1111/hex.70752

**Published:** 2026-07-03

**Authors:** B. Heckemann, E. Varkey, P. Andréll, A. Wolf, R. Johannsson, P. Forslund, K. Cerná

**Affiliations:** ^1^ Department of Anaesthesiology, Intensive Care Medicine and Pain Medicine Sahlgrenska University Hospital/Östra Gothenburg Sweden; ^2^ Institute of Health and Care Sciences, Sahlgrenska Academy University of Gothenburg Gothenburg Sweden; ^3^ Department of Occupational Therapy and Physiotherapy Sahlgrenska University Hospital/Östra Gothenburg Sweden; ^4^ Department of Health and Rehabilitation/Physiotherapy, Institute of Neuroscience and Physiology, Sahlgrenska Academy University of Gothenburg Gothenburg Sweden; ^5^ Department of Anesthesiology and Intensive Care Medicine, Institute of Clinical Sciences, Sahlgrenska Academy University of Gothenburg Gothenburg Sweden; ^6^ Patient Representative Levande Bibliotek (Living Library) Gothenburg Region Västra Götaland Sweden; ^7^ School of Information Technology Halmstad University Halmstad Sweden

**Keywords:** co‐design, co‐design facilitation, patient and public involvement, reflective practice, relational work

## Abstract

**Introduction:**

Involving patients and the public in healthcare research, development and innovation is becoming mandatory. Co‐design is a structured approach to collaboratively investigating and finding solutions to complex problems that brings together relevant actors and interest groups. Including experiences and insights from patients and the public in multi‐actor co‐design processes can help to generate and evaluate solutions to complex problems. While toolkits support co‐design project organisation, little is known about the relational work required when facilitating co‐design workshops, especially in healthcare, where professionals often learn facilitation skills ‘on the job’. Reflective accounts are scarce, despite the complexity and unpredictability of co‐design. Based on reflective conversations between two co‐design workshop facilitators, this article explores co‐design facilitation as relational work and offers a conceptual model to guide reflection.

**Methods:**

Authors B.H. and K.C. facilitated five co‐design workshops involving people living with chronic pain between September 2023 and January 2024. Within 24 h of each workshop, individual reflective interviews (B.H., *n* = 5; K.C., *n* = 5) and dyadic reflective discussions between the two authors (*n* = 5) about the learning and insights gained from the workshop facilitation were recorded. The average length of the interviews and dyadic discussion was approximately 18 min. The data were automatically transcribed and analysed in a reflexive thematic analysis.

**Results:**

Facilitation required managing unpredictability alongside structural considerations. Three interconnected themes were identified: (1) the planning, evaluation and learning space; (2) the workshop‐holding space, comprising structural, emotional, physical and activation subspaces and (3) the tacit space. We present an overarching theme and conceptual reflection model: ‘Holding complex spaces through relational work’.

**Discussion:**

Findings show that co‐design facilitation is demanding relational work that involves continuous interpretation and awareness. The conceptual model provides a flexible reflective tool for understanding facilitation complexity, rather than a procedural guide.

**Conclusion:**

This case study shows that co‐design facilitators must hold complex, unpredictable relational spaces. While toolkits offer useful structure, facilitation hinges on navigating relational dynamics. Our conceptual reflection model provides a flexible way to understand this complexity through interconnected spaces and to support reflective skill development. Developed in chronic pain contexts, the model may be transferable to other healthcare settings; future work should examine its value for facilitator training in co‐design.

**Patient or Public Contribution:**

Two patient representatives are co‐authors of this publication and provided critical input on the findings and their interpretation. Both have been co‐researchers in the reported co‐design project since its inception and contributed throughout study planning, workshop delivery, the reflective analysis and reporting of results in the current study.

## 1 Introduction

Patient and Public Involvement (PPI) has become a requirement in healthcare research, innovation, governance and management [1, 2, 3]. The aim of PPI is to ensure that treatments and services are designed to match patients’ needs.This requires that research is being carried out in collaboration, that is, ‘with’ or ‘by’ members of the public rather than ‘to’, ‘about’ or ‘for’ them. The UK Standards for Public Involvement are a framework that describes underlying PPI principles, including valuing all contributions, mutual respect and productive relationships suitable for working together for a common purpose [4].

PPI and co‐design are closely connected and aligned through shared values. Both emphasise the importance of bringing together relevant actors for service development, improvement or innovation and healthcare research [5, 6], while being rooted in democratic principles that empower end users through collaborative problem‐solving [5, 7].

As PPI is becoming mandatory, co‐design provides methodologies to translate PPI into practice [8, 9]. Co‐design methodologies may be applied at the micro level, for example to improve local service quality and access, through to the macro level, where they inform governmental policy and organisational strategy [10]. In healthcare research, co‐design is frequently used to set research agendas and refine study plans [11].

Co‐design methodologies encompass a range of methods, including observations, individual interviews and focus groups, with interactive workshops serving as the primary format for collaboration [12]. Their purpose is to bring together relevant actors for hands‐on collaboration and problem‐solving, often using supportive tools and creative activities [13]. Commonly used tools include visualisations, brainstorming, or storyboards. However, as each co‐design project has different stakeholders and aims, there are no specific methods or set curricula. Each workshop needs to be purpose‐designed and tools and activities adapted to suit workshops’ aims and participants [14]. Many toolkits that guide co‐design processes are freely available online. They support co‐designers in structuring workshops, choosing activities and organising entire co‐design projects [9, 13, 15, 16]. However, most toolkits are generalised templates that are process‐ and methods oriented, and some authors critique this emphasis on tools and mechanistic processes over human relationships in co‐design [17, 18].

Indeed, human relationships are an essential component in co‐design. The success of a co‐design workshop depends on the participants’ ability to collaborate effectively, to overcome their differences, and to establish mutual trust. Assembling a group of relevant actors in one place is not sufficient for co‐design to happen [19]. Skilled facilitation is crucial in bridging diverse perspectives, mutual learning and fostering meaningful connections among participants [20].

However, the role and practices of co‐design workshop facilitators are poorly understood at present. Workshop facilitators are a heterogenous group, encompassing people from diverse professional backgrounds [21, 22, 23]. In healthcare, facilitation roles as educator, coach and in implementation projects are well established [24, 25]. Facilitating co‐design is a more recent role for healthcare professionals that requires different skillsets, including enabling collaboration and guiding diverse stakeholders through complex, often messy problem‐solving processes [23]. Traditionally, professionally trained designers facilitate co‐design processes, and healthcare professionals usually lack this training. However, with co‐design becoming increasingly mandatory, healthcare professionals might have to ‘learn on the job’. Numerous toolkits are available to support co‐design even for inexperienced staff, and, as the Agency for Clinical Innovation states on their website: ‘True co‐design in healthcare is hard—something is better than nothing’ [13]. However, unskilled facilitation might not yield optimal results. Effective facilitation is cognitively demanding [26], as it requires sensitivity to recognise and guide both the group and the individual to ensure that people develop rapport, understand and trust each other to learn and design collaboratively [27]. Facilitators can thus experience the co‐design process as uncomfortable, challenging and demanding [27, 28].

Reflection is a tool for analysing actions, decisions and outcomes to develop an understanding for both the self, as well as the current and completed work, and to inform future work. Reflection is thus an iterative process that can be applied before (*for action*), during (*in action*) and after (*on action*) co‐design workshops. The aim of reflection is to broaden experience and to acquire a larger repertoire of skills, information and techniques [29]. Reflection can help workshop facilitators to enhance their skills and ability to feel and cognitively process the group dynamics during the co‐design process [17]. It can thus bridge the gap between workshop methods and skillful facilitation.

Despite growing interest in the co‐design facilitation experience [23, 30, 31], empirical studies that examine the facilitator experience are scarce. This case study addresses the gap by analysing reflective facilitator accounts recorded after each of five sequential co‐design workshops that included people living with chronic pain. Specifically, we examine the complex relational work entailed in co‐design [17, 23]. Drawing on the findings, we offer guidance for reflection on the complex interpersonal dynamics inherent in healthcare co‐design.

## 2 Aim

Based on reflective conversations between two co‐design workshop facilitators, the aim of the study was to: (a) explore co‐design facilitation as a relational work and (b) offer a conceptual model to guide reflection.

## 3 Context

**Figure 1:**
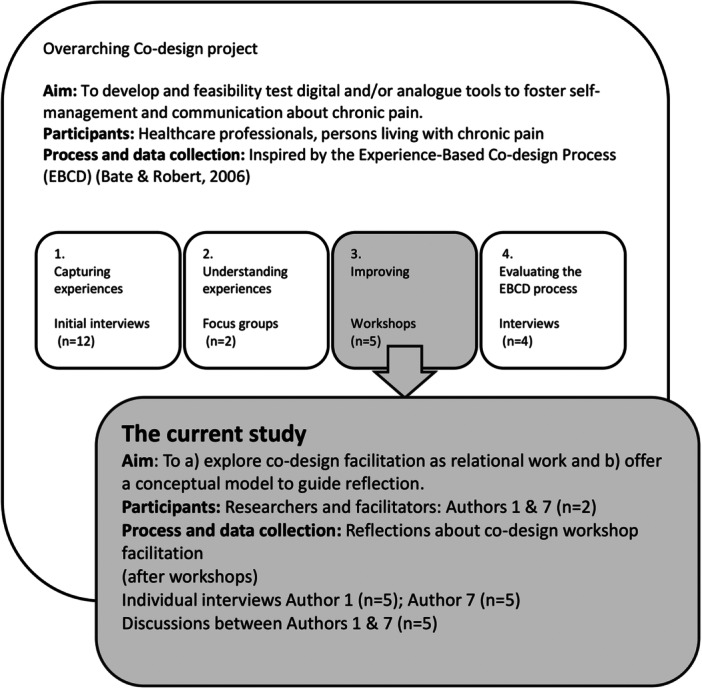
Overview of the overall co‐design project and the position of the present study, including aims, participants, process and data collection.

This study is embedded in a larger research and innovation co‐design project that is based at a Specialist Pain Clinic in Sweden. The Specialist Pain Clinic provides pain care for people living with chronic pain throughout the entire region, as part of the state‐funded Swedish national health service. The project is inspired by the four steps of the *Experienced‐based co‐design* (EBCD) approach [8]. The overall project aims at developing and feasibility‐testing analogue and digital tools to support person‐centred communication and care planning between persons living with chronic pain and healthcare professionals [32]. Figure 1 situates the present study within the overall co‐design project and summarises the aims, participants, process and data collection for both the present study and the wider project.

### 3.1 Description of the Workshops

We conducted five co‐design workshops (four in‐person, in private meetings rooms on the hospital premises, one online due to weather conditions) between September 2023 and January 2024, scheduled with approximately 4‐week intervals.

In preparation for the workshops, B.H., R.J., P.F. and K.C. collaboratively developed a preliminary plan with topics for each workshop in the series. The workshops followed an iterative design: topics were updated, considering the results from the preceding workshop. The team met for detailed planning 2 weeks before each workshop, adapted the topics if needed, and discussed the activities.

Each workshop lasted about 90 min, including a break, and followed a consistent structure (Table 1). This included a welcome section comprising a short grounding meditation for participants to ‘arrive’ in the room and a reminder of the ground rules for the workshops, which had been collaboratively agreed upon in the first workshop. A sharing round about how participants felt and what was important in that moment followed the welcome section. To support the sharing narrative, participants were provided with wooden mannequins and soft materials like strings, fabric and ribbons. Participants could put the mannequin into a shape and decorate it to reflect their state. The sharing round was followed by another creative activity or discussion round to explore the specific workshop topic. The workshop ended with a summary of the results and final reflections.

**Table 1:** Basic workshop structure template.

Activity	Description	Time (min)
Welcome	Short meditation to land in the workshop, presentation of agenda and ground rules	10
Sharing round: Where am I here and now?	Explorative work with wooden mannequins, body maps	20
Main activity (incl. coffee break)	Introduction of activity to explore workshop topic. This could be creative activities such as designing a diary page, and/or discussions	45
Rounding up	Exploration: Where am I here and now. Summarising findings and final reflections	15

The composition (facilitators, participants) for each workshop is detailed in Table 2. B.H. and K.C. served as the primary facilitators for all five workshops. The workshop participants living with chronic pain, that is, pain lasting longer than 3 months, were adult Swedish‐born persons. They had different primary diagnoses including Ehlers‐Danlos syndrome (*n* = 2), chronic arthritis (*n* = 2) and chronic pain following whiplash trauma (*n* = 1). Four participants were female; one was male. Three participants (two women, one man) attended all five workshops. One woman attended only the second workshop, another woman who had attended workshops 1–4 felt unable to attend the final workshop. Participants had been recruited via the Living Library, a database within Region Västra Götaland that establishes contact between patient representatives and research teams (*n* = 3) or via word of mouth (*n* = 2). All participants had been living with chronic pain for decades and had ample experience with various types of healthcare professional (physiotherapists, physicians and nurses) when seeking help for their pain. Three participants also had ample experience in peer‐support and peer‐teaching activities.

**Table 2:** Workshop topics and participants.

Workshop number (Date)	Topic	Participants		
		Persons living with chronic pain (participant)	Researchers/facilitator (B.H. and K.C.)	Healthcare professional (participant)
#1 (14 September 2023)	Exploring the narrative	*N* = 4	*N* = 2	*N* = 1
#2 (16 October 2023)	Diaries	*N* = 5	*N* = 2	—
#3 (12 November 2023)	Communication with healthcare professionals	*N* = 4	*N* = 2	—
#4 (07 December 2023)	Strategies/having needs met	*N* = 4	*N* = 2	—
#5 (25 January 2024)	Needs and wishes for digital tools	*N* = 3	*N* = 2	—

Only the first workshop was attended by a healthcare professional (physiotherapist) as a stakeholder participant. Due to recruitment difficulties at the Specialist Pain Clinic (high workload and lack of time to participate), the team decided to continue the workshops without inclusion of healthcare professionals after Workshop 1.

## 4 Facilitators’ Positions and Backgrounds

K.C. holds a PhD in pedagogy and has 8+ years’ experience of working with participatory approaches, including co‐design. She is an expert in co‐design/human‐computer interaction and worked on several projects which used co‐design to develop tools supporting people with chronic conditions.

B.H., the principal investigator, is a registered nurse and senior researcher focusing on qualitative research methods, PPI, yet new to workshop facilitation in co‐design. B.H. had theoretical knowledge of co‐design and followed K.C.'s lead in learning about the practicalities of co‐design.

While K.C. comes from a background of participatory co‐design, which includes a strong focus on power‐sharing and collaborative learning, B.H. approached the co‐design process from an action‐oriented position. During the reflections, the differences in positionality became evident. They enriched the discussions and reflections, fostering learning about co‐design for both facilitators.

## 5 Ethical Considerations

The current study was exempt from overall ethical approval (Swedish Ethical Review Authority, Dnr 2022‐03452‐01 and Dnr 2023‐02249‐02). The data were generated through B.H. and K.C.'s own self‐reflection and introspection through conversations; no external participants were included. The reflections were voluntary, and the researchers retained full control over their personal information.

## 6 Materials and Methods

### 6.1 Design

In this qualitative descriptive study, we audio‐ and video recorded and transcribed interviews and discussions about the co‐design processes between two workshop facilitators (B.H. and K.C.). We used reflexive thematic analysis [33] to systematically analyse the transcriptions. The data were interpreted into a conceptual model.

### 6.2 Data Collection

Between September 2023 and January 2024, individual reflective interviews (B.H., *n* = 5; K.C., *n* = 5) and facilitated dyadic reflective discussions between B.H. and K.C. (*n* = 5) were conducted. The interviews and reflective discussions were scheduled as debriefing sessions no more than 24 h after the co‐design workshops to ensure that B.H.'s and K.C.'s memories of the co‐design workshops were still fresh [34].

On each appointment, a moderator (G.B.) interviewed B.H. (*n* = 5) and K.C. (*n* = 5) individually. Subsequently, the moderator facilitated dyadic reflective discussions between B.H. and K.C. (*n* = 5). The interviews and discussions lasted between 15 and 20 min each; the average duration was approximately 18 min. All interviews and discussions were in English, video‐ and audio‐recorded using Zoom video conferencing software (Zoom Communications Inc., San Jose, CA, USA) and automatically transcribed using Microsoft Word for Microsoft 365 (Microsoft Corporation, Redmond, WA, USA). B.H. checked the transcripts for quality and correctness. ATLAS.ti Web (version 8.3.0, ATLAS.ti Scientific Software Development GmbH, Berlin, Germany). G.B. typically asked questions about each workshop concerning the most relevant (both challenging and fruitful) moments and the learning gained from the experience:

- Can you describe the richest or most important moment of the workshop?
- Can you describe a challenging moment?
- What did you learn as a facilitator?

## 7 Use of Artificial Intelligence

We used ChatGPT (GPT 5.2 Instant, OpenAI, San Francisco, CA, USA) as a writing tool to improve the readability of text passages, suggest more concise phrasing and support proofreading. All AI‐assisted text was reviewed, verified and edited by B.H., who takes responsibility for the accuracy, originality and references in the manuscript.

## 8 Data Analysis

We conducted a data‐driven, reflexive thematic analysis to analyse transcribed material [33]. Braun and Clarke describe an iterative, inductive process that commences with coding, followed by theme development. In the final phase, the themes are conceptualised and organised as per a central organising concept. Typically, the process includes six iterative steps: familiarisation; coding; generating initial themes; reviewing and developing themes; refining, defining and naming themes; and writing up.

B.H. and K.C.'s pre‐understanding and the interview questions about learning, facilitation, and co‐design guided the inductive code generating process. Due to their distinct backgrounds, B.H. and K.C. had different preconceptions of the data. While B.H. was more clinically and outcome‐oriented, K.C. focused more on power dynamics and participatory processes.

Both authors immersed themselves in the material and coded broad meaning units in a first coding cycle. While B.H. was the main coder, B.H. and K.C. met frequently to discuss the coding, to generate themes and to construct the overarching theme.

### 8.1 Triangulation

The themes were presented, discussed and finalised in a meeting with authors B.H., E.V., P.A., R.J. and P.F. to ensure their relevance to both clinicians and patients. P.F. participated in four, R.J. in all co‐design workshops, and they were able to corroborate and challenge the analysis from their perspectives and experience of the workshops. E.V. has experience as a participant in other co‐design projects. P.A. contributed from her perspective as a specialist physician in pain medicine, as a participant in other co‐design projects, and as a senior researcher. Table 3 shows the final coding scheme including themes, definitions and subthemes.

**Table 3:** Final coding scheme.

Theme	Definition	Sub theme
1. The planning, evaluation and learning space	Planning, evaluation and organisation (pre‐ and post‐workshop)	1.1 Weighing process versus outcome, 1.2 Setting goals and agenda, 1.3 Choosing (venue, resources, activities, equipment), 1.4 Debriefing and digesting
2. Holding the workshop space	Facilitating the workshop	2.1 The structural space (following agenda, managing technology), 2.2 The emotional space (providing emotional safety), 2.3 The physical space (room, equipment), 2.4 The activation space (balancing emotional, physical and structural space)
3. The tacit space	Acknowledging unexpressed emotions, experiences and motives	

## 9 Rigour: Trustworthiness

Discussing trustworthiness is especially important in this study, as the researchers B.H. and K.C. studied their own experiences as workshop facilitators, which entails the risk of limited perspectives. We ensured trustworthiness (credibility, transferability, dependability and confirmability) [35] by reflecting individually and in discussions on experiences with the workshop facilitation shortly (max 24 h) after running the workshops. Our memories were still fresh at this point, which reduced recall bias (credibility). Furthermore, we endeavoured to provide clear descriptions of the context of the workshops to enable readers to judge whether our findings are applicable to their respective contexts (transferability). Finally, we discussed our findings with our co‐researchers, some of whom had been involved in the workshops, while P.A. had a more neutral stance, as she had not participated (dependability and confirmability).

## 10 Results

This reflexive thematic analysis [33] of transcribed material generated three themes, eight subthemes (Table 3), and one overarching theme. In the following, we explain the themes and their relationships and provide illustrative quotes from interviews and discussions.

**Figure 2:**
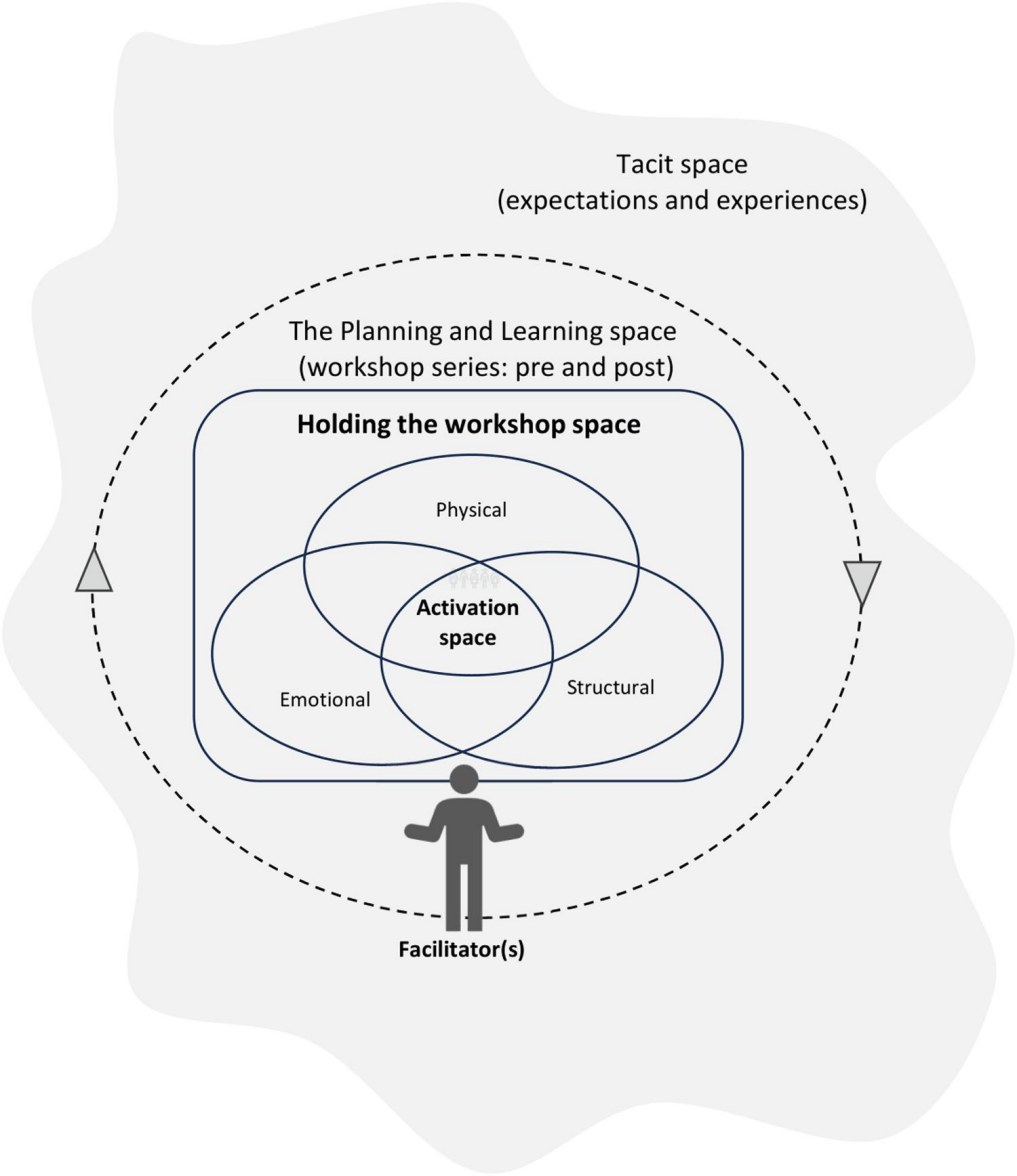
Conceptual model: Holding complex spaces through relational work.

The overarching theme ‘Holding complex spaces through relational work’ (Figure 2) positions the facilitator as a ‘spaceholder’ throughout the co‐design trajectory. Our interpretation emphasises that the trajectory is sustained by continuous relational work across three spaces (themes): (1) Planning, evaluation and learning space; (2) acting: holding the workshop space and (3) acknowledging: the tacit space (Cf. Table 3 for the final coding scheme).

### 10.1 Theme 1: The Planning and Learning Space

*The planning, evaluation and learning space* was the most explicit space of the co‐design trajectory because it entailed all activities that happen on ‘the surface’: the planning, evaluation and goal setting. Beyond the planning, it facilitated meta discussions between B.H. and K.C. about underlying assumptions. The collaboration between facilitators and patient co‐researchers (R.J. and P.F.) required relational work from everyone to ensure open, honest communication and learning, as well as reconciliation of differing agendas.

Weighing process versus outcome

The meta discussions revealed tensions between KC's focus on moments of co‐creation and learning and BH's focus on outcomes.

What I can say is that the co‐design process is extremely messy, I […] was a bit naive and thought it [the workshop] would just go as per plan, but it's not going at all according to plan […].

(B.H., Individual Interview (IV), WS 3)

[…] the question was actually “where am I here and now?” And it kind of became something bigger for everybody. And I got slightly impatient because it dragged on for such a long time. It was really meant to be an icebreaker […] and it just became something bigger.

(B.H., Discussion WS 3)

You know, this was like uh, this is like the very kind of creative or like a participatory part that is basically like a topic we haven't planned for. And it emerges through the conversation and through it requires kind of us as organizers of the participatory process, to open up and let go and say, OK, there is a new idea coming up. We take it instead [of the initial idea/plan].

(K.C., IV, WS 4)

1.1. Setting goals and agenda

To ensure relevance, B.H., R.J., P.F. and K.C. collaboratively designed the workshop agendas. This structure was iterative, allowing topics to be adapted after each session to better reflect the evolving needs and insights from the preceding workshop.

1.2. Choosing (venue, resources, activities and equipment)

B.H., R.J., P.F. and K.C. discussed and chose the activities, environment, and necessary equipment in preparation for each workshop.

This is something to also include into the planning of the workshops […] we just have to make sure that even a simple thing [like the right chair] is in place. What type of chair you're bringing can actually play a role for someone to be able to take part fully.

(K.C., IV, WS 3)

K.C.'s expertise in participatory design was essential for choosing relevant activities; together, B.H. and K.C. created individual portfolios for workshop participants. The portfolios featured worksheets for reflection, note‐taking, and creative brainstorming activities, alongside copies of each workshop's slide show. To ensure they remained current, the team kept these folders on‐site, allowing for the iterative addition of new materials as the curriculum evolved based on participant feedback. B.H. and K.C. also prepared icebreaker and relaxation activities, or activities that enabled participants to speak and reflect about their present moment experience through body maps or wooden mannequins that were decorated with ribbons and fabric to support sharing participants’ current state. These activities served as tools to support the relational work during the workshops.

1.3. Debriefing and digesting

Team meetings served as a space for collective reflection and debriefing and allowing the team to process emotions. The discussion topics ranged from addressing initial apprehension to navigating the frustration caused by the inherent unpredictability of the co‐design process.

I had a bit of problem with fixing the camera and the recording equipment. […] That's part of what made me nervous.

(B.H., IV, WS 1)

Because there was so much discussion going on and there was also some distraction, […] one participant had to get up and walk around, had to take a pill, a phone call. So, there was disruption […] I learned more […] about the topic [chronic pain] than about doing the research in a practical way.

(B.H., IV, WS 3)

### 10.2 Theme 2: Holding the Workshop Space

We defined ‘holding space’ as the relational work of maintaining a safe environment where participants could share their experiences without fear of judgement or interruption. This required high levels of flexibility and self‐awareness; B.H. and K.C. had to attune to the group's needs and engage in the co‐process without becoming fully immersed in it. We identified three interrelated spaces: the structural, the emotional, and the physical space. In the following, these are described in detail.

2.1. The structural space

The structural space encompasses the workshop agenda, technology (recording equipment, presentations) and preparation of the ‘fika’ (Swedish coffee break). Planned activities, such as meditation, drawing, and writing provide a scaffold for the workshops. For example, using wooden mannequins draped in ribbons or fabric helped elicit participants’ current emotional states, supporting personal narratives and fostering empathy within the group:

One person, for example, she bound it [the mannequin] with this red paper ribbon [at the beginning of the workshop]. And she said at the end [of the workshop], “I'm going to take it off”. […] At the beginning she was using it to communicate to us how she's feeling and what she's experiencing […]. The figure kept on falling down and she used it [the falling down] as an illustration. “This is how it is for me right now. I just need to lie down”. It was really working well; the figures could communicate something […]. And one of the participants also kind of like petted it [the mannequin], [saying] “there, there” as a kind of expression of: “you need this” or “you need this kind of pat on the shoulder” or something like that. So, they [the mannequins] were playing a big role today.

(K.C., IV, WS3)

2.2. The emotional space

While participants were motivated by a genuine desire to improve healthcare, they also brought lived experiences that were frequently emotionally charged. As K.C. noted:

There's something they have experienced in the past that's very strong for them. And then they kind of bring it into the design context […] they feel like, ohh I want to share this now. But […] it's not always relevant to the design process.

(K.C., IV, WS 2)

Accordingly, managing the emotional space required sensitivity to individual personalities and often subtle behavioural signals within the group.

I was thinking about one of the participants closed her eyes when someone else was talking, which is a common strategy when people feel a bit overwhelmed and maybe feel like it's too much input.

(K.C., interview, WS3)

B.H. and K.C. had to simultaneously manage workshop activities while balancing the tension between addressing the group's emotional needs and achieving the session's specific design goals.

I mean, usually the meditation at the beginning makes everybody land in the room and just ready to work. And this time it just really brought up emotions. […] There was space for that, and we just honoured that with some silence. But we could not sit in there for too long because we had a workshop and obviously my aim is to get somewhere [i.e., achieve the workshop's goal] while still accommodating people's needs.

(B.H., Discussion, WS 5)

2.3. The physical space

Adapting the physical space to participant needs is essential relational work; physical comfort and attunement enable participants to contribute to the best of their ability.

…it's very common during workshops that people are sitting down and for people who suffer from a lot of pain [this is] not always possible. So today, at least two people were standing up, [or] sitting down a lot because it was difficult for them to sit down. And this is something to include in the planning of the workshops, to make sure that there is place and space for people to actually stand up. […] sometimes it's not comfortable for them sitting in certain type of chairs […].

(K.C., IV, WS 3)

2.4 The activation space

Co‐creating is the raison d’être of the co‐design workshop; we called this the ‘activation space’, where the group collaborated and created as one unit, rather than a group of individuals. B.H. and K.C. experienced that flexible management of activities and conscious relational work enabled the group to enter this space of organic learning and creation.

They created diary sheets and […] they learned from each other. One participant had the [Swedish] word ‘glad’, which means happy, at the bottom of his page in very big letters. And he just filled it out [colored the letters] to a certain degree. So, at the end of the day, he wasn't totally happy, but maybe he was half happy […] and I think that most participants took on board that that's a nice way of depicting how you're feeling at the end of the day.

(B.H., IV, WS3)

### 10.3 Theme 3: The Tacit Space

The tacit space comprises the unexpressed experiences and expectations held by workshop participants and facilitators. The reflections revealed the facilitators’ own assumptions and expectations. The participants came to the workshops with their own expectations and experiences, some of which were articulated, but most were probably not. Acknowledging the tacit space entails that the facilitators remained aware of and comfortable with the ‘unknown’, that which was not communicated but could influence the workshops in subtle and sometimes even disruptive ways.

[…] the patients, they bring in these very strong stories. But from my perspective, they're not always connected to the design context that we're trying to work with. But they [the participants] kind of don't care. They just want to throw it in there for whatever reason. They find it relevant. And my big question is how do we deal with that? […] From my perspective, it was just so interesting when I said this [issue the participant was raising] is not something we can tackle. And X [the participant] was like, I don't care. I want to tell you anyway.

(K.C.,Discussion, WS2)

[RESPONSE B.H.]: I think it was interesting that this happened towards the end of the workshop. We had run over time, which wasn't good. And Y [participant] also said ‘I can't do this anymore. It's too much for me’. I think, because X kept saying ‘I don't care’.

(B.H., Discussion, WS2)

The tacit space can be framed as an additional relational dimension in the co‐design process. Tacit assumptions, norms, experiences, or agendas show up in observable behaviours and patterns of participation, for example, attempts to dominate or side‐channel the discussion, defensiveness or disregarding the stated and agreed‐upon ground rules.

[…] then this moment between A and B when B was describing that her physiotherapist knows her better[than she knows herself]

B.H. and K.C. agreed that the tacit space is best managed with compassion, treating its expression as data or information. Instead of asking ‘why’ a person was acting the way they were, they focused on ‘what’ happened to the group dynamics and aimed to steer the group in a direction that was conducive to the co‐design process.

Then again, the stories. So, this has been an ongoing issue […]. People always want to share these stories with us […] it's interesting for me […] we asked about this app [testing an app for pain management] and how it worked for them, and they tell us about how will they need to have a certain [short] haircut? For like an outsider, this is completely irrelevant information, but for them [the participant] it makes sense. But I think it's important to remember that we're trying to understand how they make sense of their pain experience. And the way they make [this] experience through their pain is through their life, because that's what it is.

(KC, IV, WS5)

## 11 Discussion

Based on the reflexive conversations of two co‐design facilitators (B.H. and K.C.), this study identified three spaces that require relational work: (1) planning, evaluation, and learning space; (2) holding the workshop space; (3) the tacit space. We integrated our findings into an overarching theme: *Holding complex spaces through relational work*.

In the following section, we will discuss the relational work of *holding complex spaces* to foster an understanding collaboration in co‐design work, above and beyond template‐style mechanistic approaches.

### 11.1 Holding Complex Spaces

We intentionally employ the metaphor of ‘space’ and the overarching theme of ‘holding space’ to illustrate the complex nature of co‐design facilitation. In design research, space is viewed not merely as a physical setting or room, but as a resource and opportunity defined as the interplay between physical environments and social relationships [36, 37]. Within co‐design, facilitators strive to cultivate safe, nurturing spaces, as described by Cerna et al.'s concept ‘situated scaffolding’. Situated scaffolding entails providing temporary, empowering support for workshop participants [38]. However, even well‐structured spaces can be sites of tension where participants experience stress, which can cause emotional challenges for the facilitators [39].

We contribute to this literature by acknowledging that the inherent ‘messiness’ of co‐design precludes a rigid, controlled co‐design process that rests on the rigorous application of methods. Instead, we propose a responsive approach: holding complex spaces. This allows facilitators to use methods and tools to create scaffolding or structures, while maintaining responsiveness to participant needs. In this case study, the *planning, evaluating and learning space* enabled us to intentionally design *the structural* and *physical spaces* that catered for participants’ needs and guided the workshop towards the envisioned outcomes.

The *emotional space* was the least controllable space; however, activities and design games can be used as tools to enable dialogue and create structures that support idea generation and collaboration by tapping into a certain playful mindset [40]. Facilitators should therefore prioritise using artefacts and engaging, playful co‐design activities into their workshops over heavily cognitive discussions. This requires facilitators to be creative; a ‘playful mindset’ cannot be reduced to a standardised method. A playful mindset emphasises the exploratory, imaginative, dialogical, and empathic dimensions of co‐design [41].

Our results show that the three spaces are inherently interdependent: the strategic insights from the planning, evaluation, and learning space provide insights for the physical and structural spaces, which can provide the safety structures that support the unpredictable emotional space. This interdependence suggests that co‐design success relies less on a standardised application of methods than on the facilitator's ability to remain present and responsive [17]. By shifting focus from a methods‐focused process to the active ‘holding’ of these interdependent spaces, facilitators can manage the complexities of co‐design.

### 11.2 Relational Work in Co‐Design

The shift from a method‐focused process to the active ‘holding’ of these spaces fundamentally relies on relational work. Recently, design scholars have criticised over‐reliance on tools, calling instead for a ‘relational design’ approach. Nielsen and Bjerck [18] argue that the ability to act meaningfully in design emerges from ‘emotional relatedness’: social relations unfolding across time and space, rather than through mechanistic application of co‐design tools and methods [21]. The relational perspective is particularly important in healthcare co‐design, where facilitators must balance project objectives with the broad lived experiences of patients [42].

Our study advances this line of research by demonstrating that relational work is a subtle, nuanced form of human relating that permeates both the explicit and tacit spaces of co‐design. Such work is often invisible and can only be uncovered through careful, on‐action reflection [29] as presented in this study.

Indeed, team reflection ‘for’, ‘in’ and ‘on action’ [29] appears to be an essential process in view of the emotional and relational complexities of co‐design. We found that facilitators’ ability and capacity to ‘do relational work’ is paramount, as emotional and group dynamics determine participant engagement and workshop outcomes. However, ‘holding space’ for these emotions is a dual burden: facilitators must attend to participant interactions while simultaneously regulating their own internal emotional states [43]. Facilitation can elicit a broad range of emotions in facilitators, including anger and sadness in response to project challenges and disruptions, while fear was linked to unexpected events and the responsibility of keeping the workshop on track. In contrast, joy, love, satisfaction, and astonishment were associated with participant engagement and achievement [43].

Our findings are aligned with Soto Hormazábal et al. who emphasise a critical gap in current co‐design guidance: the need for expanded frameworks, tools and training specifically designed to support facilitators in managing their own emotional health and relational capacity throughout the co‐design trajectory [43]. Since workshop facilitators can come from diverse backgrounds [21, 22, 23], they may be unprepared to manage boundaries and emotional dilemmas that come with co‐design. These aspects are currently not adequately recognised and discussed in the scholarly literature, and further exploration of the emotional and potentially traumatic impact on facilitation is needed.

Furthermore, co‐design is often complicated by participant trauma. In healthcare, trauma frequently accompanies chronic illness, stemming both from the diagnosis itself and from experiences with healthcare services [23, 44]. While co‐design workshops are not therapeutic environments, they may incur emotional vulnerability and potential re‐traumatisation, raising questions regarding the facilitators’ boundaries, responsibilities, and obligation to provide support in managing trauma [23]. The co‐design community increasingly acknowledges the frequent occurrence of trauma and its challenges. While conclusive practical strategies are not yet available, trauma‐informed approaches and facilitation skills have in recent years received scholarly attention [23, 45].

### 11.3 Towards a New Conceptual Model

As a first step towards uniting mechanistic and relational work, we propose a conceptual model that serves as a reflective aid to explore the fluid relational, emotional, and structural dynamics of facilitation (Figure 2). The proposed model is the first to guide reflection on the relational aspects of co‐design, empirically grounded in a case study, and, as such, potentially filling a gap for reflection on small‐scale local co‐design projects. An alternative model, the HEC model (human‐perspective [H], experiential [E] and creative [C]) [21] includes a human dimension but provides limited guidance on the lived complexity of facilitation. Moreover, this model was designed using two large‐scale cases including multi‐stakeholder events. It may thus be too advanced for smaller‐scale, local co‐design projects.

The authors suggest using the model proposed in this paper for reflection guided by a strengths‐focused reflection style. This deliberately emphasises existing resources, capabilities, and positive experiences over concentrating on problems or deficits to support facilitator empowerment, motivation, and constructive learning about co‐design facilitation [46]. A recent publication provides complementary, practical techniques and questions that foster learning from success and exploring how challenges were overcome that can inform and inspire facilitators seeking to structure fruitful reflection in their own workshops [47].

### 11.4 Specific Recommendations

Holding complex spaces requires constant balancing of physical, structural, and emotional needs. The emotional space is particularly demanding, as it necessitates the simultaneous management of one's own internal state and the evolving dynamics of the group. Our findings suggest that the primary mechanism for managing these emotions is presence. Rather than merely reacting to events, staying present allowed us to consciously scan for subtle cues—changes in facial expressions, body language, and the overall ‘energy’ of the room. This was facilitated by a deliberate slowness, utilising silence and patience to ensure participants felt fully heard.

While we experienced no explicit conflict, we did encounter tensions, particularly when participants voiced concerns beyond the project's scope and aims. In these moments, we balanced clear transparency with validation: we clearly communicated the project's limitations while intentionally allowing participants the space to share their concerns. By permitting ‘outside’ topics to take up space, we addressed the inherent power imbalance of co‐design, ethically prioritising the participants’ right to define what was meaningful to the process.

## 12 Limitations

While co‐design facilitation is often discussed in practical terms, the relational work involved has received limited attention. Our study addresses this gap, though several limitations apply.

First, the analysis is grounded in B.H. and K.C.'s personal reflections on the facilitation experiences. They acted both as researchers and as study participants. While we acknowledge its subjectivity, self‐reflection remains a well‐established form of inquiry in complex professional settings [29].

Since this work is about subjective accounts, further research in different contexts is needed to confirm, highlight differences or complement our findings, and to assess their generalisability.

Finally, the dataset is limited to a small number of workshops and the reflections of two facilitators, which constrains the breadth of the findings. Each workshop was a unique experience, and, as per their nature, no co‐design workshop is reproducible. However, we identified common challenges that pertained to all five co‐design workshops, and which were reviewed and corroborated by co‐authors (a specialist physician, a physiotherapist, and two patient representatives), all with co‐design experience. This helped to interpret the results from multiple perspectives, which strengthens the credibility of the analysis.

We propose a conceptual reflective model and a clinical facilitation and reflection guide including vignettes based on our workshop experiences (File S1). A criticism of our conceptual model may be that it is simply a visual rendering of our interpretation. Yet, in reflexive thematic analysis, overarching themes represent higher‐order interpretations [33, 48], and we argue that it may therefore serve as conceptual reflection model. The model's permeable boundaries reflect the fluid nature of relational space. As a reflective aid, it invites open, interpretive, and experiential reflection rather than being a procedural checklist. However, the model has not been empirically tested, and further studies are needed to assess its practical usefulness. Furthermore, the model was developed based on workshops with people who live with chronic pain. However, it may be transferrable to other populations living with chronic conditions, as their life‐experiences are similar, despite differing diagnoses [49].

## 13 Conclusion

This reflective study shows that co‐design facilitators must hold complex spaces, that is, they experience considerable unpredictability when conducting workshops. Freely available toolkits offer valuable support, but co‐design facilitation is at its core relational work that requires facilitators to pay close attention to the behavioural patterns that shape the group dynamics. Furthermore, facilitators need to be aware that co‐design is about mutual learning, it is not a therapeutic space. It is important to work as a team of facilitators to strengthen all phases of the co‐design process, from jointly planning activities, to sharing roles and perspectives during the workshop, and finally reflecting together on what happened, what worked, and where facilitators can grow their competency. Our conceptual reflection model provides a flexible tool for exploring the complexity of co‐design workshops through the metaphor of interconnected spaces and for supporting reflective development of facilitation skills. Although developed in the context of co‐design workshops with patients living with chronic pain, the authors’ experiences and the resulting reflective model may well be applicable to co‐design with other populations within healthcare.

Future work should examine how the model can inform facilitator training and support co‐design in different settings and patient populations.

## Author Contributions

**B. Heckemann:** conceptualisation, investigation, funding acquisition, writing – original draft, methodology, validation, visualisation, writing – review and editing, formal analysis, project administration, data curation, supervision, resources. **E. Varkey:** writing – review and editing, validation, formal analysis, conceptualisation. **P. Andréll:** writing – review and editing, validation, formal analysis, conceptualisation. **A. Wolf:** writing – review and editing. **R. Johannsson:** writing – review and editing, validation. **P. Forslund:** validation, writing – review and editing. **K. Cerná:** conceptualisation, writing – original draft, validation, writing – review and editing, formal analysis, data curation, methodology.

## Conflicts of Interest

The authors declare no conflicts of interest.

## Supporting information

Supporting File

## Data Availability

The data that support the findings of this study are available from the corresponding author upon reasonable request.
