# Supplementary material for: Holding Complex Spaces Through Relational Work: A Qualitative Descriptive Study of Co‐Design Facilitation
Source: Health Expect. 2026 Jul 3;29(4):e70752. doi: 10.1111/hex.70752 (PMC13332330; doi:10.1111/hex.70752)
Supplement: Supplementary file 1 — Supporting File [file HEX-29-e70752-s001.pdf]

Holding complex spaces through relational work

Facilitation and reflection guide for clinicians and novices to co-design

This guide supports clinicians and novice co-design facilitators in holding complex workshop situations through relational work. It focuses on behaviours, comments, and outputs, and on small, reversible adjustments to workshop conditions that can expand activation space.

It is intended to complement existing co-design toolkits; it is grounded in our facilitation experience, has not yet been formally evaluated, and will be refined through further development and use.

The spaces model and key concepts

**Tacit space**	Unspoken expectations/experiences that shape what feels safe; noticed through participation patterns.
**Planning and learning space**	Pre–during–post work across a series: prepare, run, debrief, iterate.
**Physical space**	Room, seating, tools, accessibility.
**Structural space**	Agenda, roles, ground rules, methods, time.
**Emotional space**	Tone, permission, dignity, respectful difference.
**Activation space**	Participation becomes workable and productive, outputs become concrete.

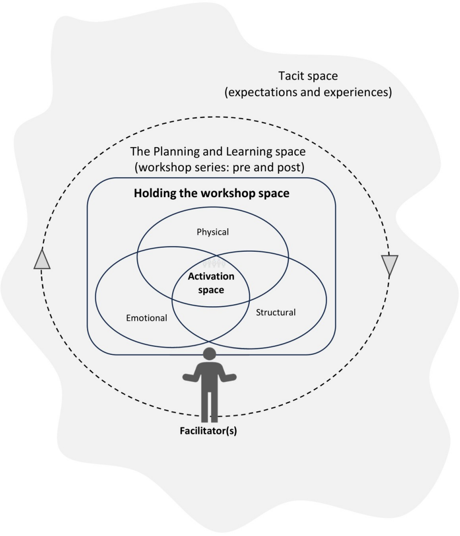

Figure 1. The spaces model.

Reflection box (use after any key moment or in debrief)

1. **Tacit space / observed cues**
   What did we see/hear (behaviors, comments, outputs, group dynamic)
   Did we treat behaviors/comments as data (not stories)? Are we maintaining non-therapeutic boundaries?
2. **Workshop space**
   What and how did we adjust (physical/structural/emotional), or not adjust in response?
3. **Activation space evidence**
   What changed (participation, learning, group dynamics, co-design output, etc.)?
4. **Planning and learning space**
   What can we learn from this workshop, what was the most important moment, what was the most challenging moment, what will we explore next time?

Worked examples (vignettes)

Each vignette is a worked example of the reflection box and comprises a brief description of a workshop situation, a facilitator move/action, a description of changes in the group dynamic and a link to the proposed model.

Vignette 1 — Start-of-session grounding (WS5)

Situation: At the opening of a January co-design session, the group's tone was subdued, low and sad. In the opening sharing round, several participants noted that their pain tends to be particularly intense during winter.

Facilitator move: We paused briefly in silence to acknowledge what had been said and to recognize the effort it took to attend. We then guided a short grounding exercise and clarified the next step in the agenda.

What changed: The room settled. Participants appeared more at ease, despite the pain, and the group was able to move into the co-design discussion and tasks.

Model link: Holding emotional space (acknowledgement/pacing) for all participants without entering therapeutic exploration.

Vignette 2 — Boundary + re-grounding (WS2)

Situation: Midway through a co-design workshop, a participant changed topic and described strongly negative experiences of healthcare. When the facilitator notes that this is outside the current focus, the participant responds, "I don't care."

Facilitator move: The facilitators paused briefly to allow the comment to land, then acknowledged it without inviting detail. They redirected attention to the shared artefact and next agenda step.

What changed: De-escalation; clearer boundary; group returned to the task.

Model link: Holding emotional (dignity) and structural (scope) space to enable activation.

Vignette 3 — Normalizing movement (WS3)

Situation: At the start of the workshop, the facilitators stated in the ground rules that participants were welcome to move around if sitting became uncomfortable. One participant attended despite having a very painful day; they frequently stood up, moved around and took pain medication during the workshop. This could have been perceived as disruptive.

Facilitator move: We treated this as expected, kept the flow; ensured that we included all participants in the activities, independent of their position in the room.

What changed: The participant could remain included without needing to explain themselves, and the group stayed focused on the co-design tasks.

Model link: Holding physical (accessibility) and structural (ground rules) space to sustain activation.
